# The Burden of Care: A National Survey on the Prevalence, Demographic Characteristics and Health Problems Among Young Adult Carers Attending Higher Education in Norway

**DOI:** 10.3389/fpsyg.2019.02859

**Published:** 2020-01-23

**Authors:** Bente Storm Mowatt Haugland, Mari Hysing, Børge Sivertsen

**Affiliations:** ^1^Department of Clinical Psychology, Faculty of Psychology, University of Bergen, Bergen, Norway; ^2^Department of Psychosocial Science, Faculty of Psychology, University of Bergen, Bergen, Norway; ^3^Department of Health Promotion, Norwegian Institute of Public Health, Bergen, Norway; ^4^Department of Mental Health, Norwegian University of Science and Technology, Trondheim, Norway; ^5^Department of Research and Innovation, Helse Fonna HF, Haugesund, Norway

**Keywords:** young adult carers, national student survey, prevalence, demographic characteristics, negative health outcomes

## Abstract

**Objective:**

The aim of the present study was to examine prevalence, characteristics and health outcomes among young adults (18 to 25 years) who provide informal care to family members or others with physical or mental illnesses, substance misuse or disabilities.

**Design:**

The sample was obtained from a national survey in Norway from 2018 among students in higher education (the SHoT2018-study). The current sample comprise 40,205 participants, 70.2% women, mean age 22 years (*SD* = 1.7).

**Outcome Measures:**

Participants answered questions on care responsibilities, mental health problems (The Hopkins Symptoms Checklist-25), insomnia (sleep questionnaire), somatic health (Somatic Symptom Scale-8), and life satisfaction (Satisfaction With Life Scale).

**Results:**

5.5% of the respondents reported having care responsibilities. Caring was associated with being female, single, having divorced parents, being an immigrant, and having financial difficulties. More mental health problems, insomnia, somatic symptoms, and lower life satisfaction were found among respondents with care responsibilities. Number of hours of caring was associated with negative health outcomes in a dose-response pattern.

**Conclusion:**

Professionals within health care, social services and the educational system should be sensitized to the needs of young adults with care responsibilities for family members or others with illness, substance misuse, or disabilities. The negative health problems among these young adult carers (YACs) should be acknowledged, and adequate support made available.

## Introduction

Young adult carers (YACs) are defined as individuals between 18 and 25 years who provide informal care, support or assistance to family members with disability, chronic illness, mental health issues, or substance misuse problems (Becker and Becker, 2008; Day, 2015). YACs are assumed to carry out substantial caring tasks, and to take on a significant level of responsibility (Becker and Sempik, 2018). The tasks performed may be practical, including household tasks (e.g., cooking, cleaning), emotional care (e.g., supporting, supervising), practical support (e.g., paying bills, administer medication), or personal care (e.g., washing, bathing, and dressing) (Becker and Becker, 2008).

The age period between late adolescence and mid-twenties (about 18 to 25 years) has been termed “emerging adulthood.” In industrialized countries, this may be a prolonged period of exploration without commitment (Arnett, 2007). However, this may also be a developmental phase where important life choices are made regarding education and/or professional career. Furthermore, this is a period for developing a more equal relationship with parents, managing financially, and establishing emotional independence (Sharon, 2015). Although many experience “emerging adulthood” as a period of personal growth, increased autonomy and maturity, others may experience this as a demanding life phase, characterized by uncertainty and challenge (Arnett, 2007). Emerging adulthood may be a particularly difficult life phase for YACs, who need to balance their time between caring and being independent, and who need to find ways to fulfill personal, social, and professional aims without neglecting their care responsibilities (Becker and Becker, 2008; Day, 2015; Care2Work, 2017). Family members and others may depend on that the YAC prioritizes his/her role as carer. It is reasonable to assume that for many YACs it is difficult to manage potentially opposing responsibilities and expectancies.

Lack of large-scale surveys makes it difficult to estimate the prevalence of young adults with significant care responsibilities. This limits the possibility to predict who among YACs are in need of support and who are coping well. As the number of research studies on YACs are limited, we lack knowledge on many aspects of the life of these young people. A recent European report indicates that we need to know more about ethnicity and financial circumstances for carers below 30 years (Care2Work, 2017). As few studies have focused on the context of caring, we have limited knowledge on specific dimensions or characteristics of the young adult carer population, such as gender, social class, family structure, financial situations or ethnicity (Aldridge, 2017). Gender has been examined in some studied with children and adolescent with care responsibilities, reporting mixed findings. Some studies indicate that girls assume more caregiving tasks than boys (Nagl-Cupal et al., 2014; Becker and Sempik, 2018; Leu et al., 2018), whereas other studies, including both young carers below 18 years and YACs, have found higher prevalence of male caregivers (Levine et al., 2005; Pakenham and Cox, 2015). Evidently, more research on the relationship between gender and caring responsibility in YACs is warranted.

Caring for others has been described as rewarding and meaningful, bringing positive emotional and psychological benefits for the carer as well as the ill or disabled family member (Haugland, 2006; Cheesbrough et al., 2017a). Having care responsibility may contribute to the development of practical and emotional skills, and is considered to stimulate resilience, problem-solving, empathy, sensitivity and ability to cope with life-challenges (Becker and Becker, 2008). However, research with children, teenagers and older adults with care responsibilities, has shown that there may also be negative outcomes on the mental and physical health of carers (Nagl-Cupal et al., 2014; Berglund et al., 2015; Van Loon et al., 2017; Kallander et al., 2018). Besides a few studies on health outcomes for YACs, we have limited knowledge about the impact of care responsibilities in this life phase. One exception is a study of undergraduate students (*N* = 353), reporting that young present and/or past caregivers (18–24 years) showed more symptoms of anxiety and depression than a control group of non-caregivers (Greene et al., 2017). Clinical levels of symptomatology was reported among many of the YACs. Another study, including both young adults below 18 years and YACs (*N* = 245, 10–25 years) reported higher somatization and lower life satisfaction in carers compared to non-caregivers (Pakenham et al., 2006). A third study, re-analyzing two surveys of samples of 18–25 year olds, found that most YACs (91.6% and 95.7%) in both samples reported being in excellent, very good or good health (Levine et al., 2005). These diverse findings indicate that we need more knowledge on the impact of caregiving on YACs.

Furthermore, to be able to support YACs, we need to understand factors that might increase potential negative health outcomes. Amount of care responsibility may be one important moderator. A relationship between level of caregiving and level of psychological stress has been found in a national health study of adults (*N* = 90.845, median age 50.5 years) in Sweden (Berglund et al., 2015). Two studies on YACs (*N* = 44, 18–24 years, *N* = 295, 14–25 years) found no association between level of caregiving and amount of psychological distress or mental health problems (Bacharz and Goodmon, 2017; Becker and Sempik, 2018). However, others have found that youth and young adults (*N* = 2474; 9–20 years) with higher amount of caregiving responsibility have poorer mental health outcomes (Pakenham and Cox, 2015). A qualitative study (*N* = 25, 18–24 years) reports that YACs do not have enough time for themselves when balancing their care responsibilities and other commitments (Becker and Becker, 2008). This makes it reasonable to assume a relationship between amount of care commitments and health problems in YACs. Due to the limited number of studies, this association needs to be examined further.

During the last 10 years the Norwegian government has taken initiatives to improve services for children and adults who are relatives of patients with physical, mental, or substance misuse problems, e.g., changes in health legislation have been made and a national guide on how to support next of kin has been developed. When parents or children have a chronic illness or substance misuse, all public health services are obliged to look after the needs of children in these families. In spite of this development, the awareness on young family carers is still low, and no services are especially targeting young carers or YACs. Hopefully, knowledge on the prevalence, characteristics and health problems among YACs, may contribute to a larger awareness, as well as better services for this group of young adults.

To sum up, our knowledge of caregiving in young adults is limited with regard to prevalence and characteristics of this population, as well as potentially negative health outcomes for YACs. Previous studies have small sample sizes, often with an explorative, qualitative design. In the present study, we include a large cohort of students aged 18 to 25 years from a national survey in Norway. The current study fills a gap in the literature by examining demographic variables (e.g., gender, ethnicity, family structure, and financial situation) as possible predictors of young adults (18–25 years) with caring responsibilities for family members or others with physical or mental illness, disability or substance misuse. As we don’t know enough about consequences of caring responsibilities for YACs, the study assesses several areas of health problems, including internalizing problems (i.e., anxiety and depressive symptoms), sleep problems and somatic complaints, as well as a positive measure of life satisfaction. As care responsibilities and other commitments in the life of the young adult may be difficult to balance and this may be a possible source of psychological distress (Becker and Becker, 2008), we also examine the relationship between the amount of caring responsibilities and health problems among YACs.

## Materials and Methods

### Procedure

The SHoT2018 study (Students’ Health and Wellbeing Study) is a national student survey for higher education in Norway, initiated by the three largest student welfare organizations [Sammen (Bergen and surrounding area), Sit (Trondheim and surrounding area), and SiO (Oslo and Akershus)]. In the SHoT2018 study, data were collected electronically through a web-based platform. Details of the study have been published elsewhere (Sivertsen et al., 2019a), but in short, the SHoT2018 was conducted between February 6 and April 5, 2018, and invited all fulltime Norwegian students pursuing higher education (both in Norway and abroad) to participate. In all, 162,512 students fulfilled these inclusion criteria, of whom 50,054 students completed the online questionnaires, yielding a response rate of 30.8%. As the current study was an investigation of “YACs,” we excluded participants aged 26 years and older, yielding a final sample size of 40,205 participants, aged 18–25 years. The average time spent answering the questionnaire was 21 min. Although a few universities and colleges allocated time in school classes allowing the student to complete the survey during a lecture, no teachers were instructed to provide support or assistance.

### Instruments

#### Demographic Information

All participants indicated their sex and age, and participants were also asked about their relationship status (response options: “single,” “girl-/boyfriend,” “cohabitant,” and “married/registered partner”), as well as their accommodation status (response options: “living alone,” “living with partner,” “living with friends/others in a collective,” and “living with parents”). Finally, participants were categorized as an immigrant if either the student or his/her parents were born outside Norway.

#### Exposure Variable

All students were asked if they had regular care responsibilities for someone with physical or mental illness, disabilities, or substance misuse (not his/her own child/children). If answering yes to this question, the students were asked how many hours they spent on a typical weekday and weekend day to help this person(s). The exact phrasing of the questions is detailed in Table 1. These were survey questions that have previously been tested for clarity among young carers (5–17 years) and their parents (Cheesbrough et al., 2017b).

**TABLE 1:** Questions used to assess care responsibilities.

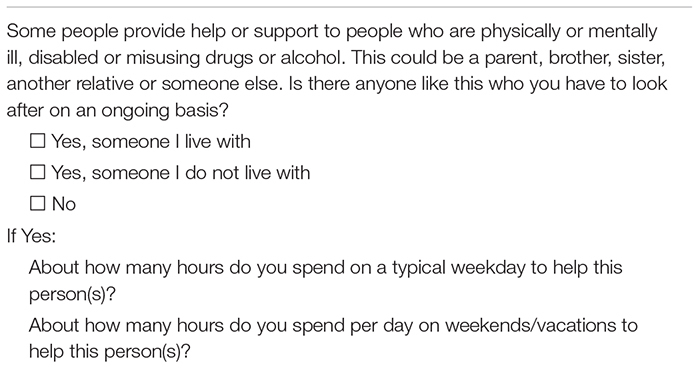

#### Outcome Variables

Symptoms of anxiety and depression were assessed using The Hopkins Symptoms Checklist (HSCL-25) (Derogatis et al., 1974), derived from the 90-item Symptom Checklist (SCL-90). This is a screening tool designed to detect symptoms of anxiety and depression. The scale consists of 25 statements regarding anxiety (10 items) and depressive (15 items) symptoms as experienced during the past two weeks, with response categories “not at all” (1) to “extremely” (4). An investigation of the factor structure of the HSCL-25 based on the SHoT-2014 data supported an uni-dimensional model in the student population (Skogen et al., 2017). Mean scores (1–4) were calculated, where a higher score indicated a higher level of anxiety and depression. In the current study, a mean score above 2.0 on the HSCL-25 was used as a conservative cut-off to indicate high levels of anxiety and/or depression.

##### Insomnia

All participants indicated the number of nights per week they experienced difficulties initiating sleep (DIS), difficulties maintaining sleep (DMS), and early morning awakenings (EMA), as well as daytime sleepiness and tiredness. Participants were then asked for how long they had suffered from these sleep problems. This information was used as an operationalization for insomnia disorder, according to the DSM-5 criteria (reports of DIS, DMS, or EMA at least 3 nights per week, in addition to daytime sleepiness and tiredness at least 3 days per week, with a duration of at least 3 months). Further details of the sleep questionnaire used in this cohort have been published elsewhere (Sivertsen et al., 2019b).

Somatic/physical health was assessed by the Somatic Symptom Scale-8 (SSS-8) (Gierk et al., 2014): an 8-item reliable and valid self-report measure of somatic symptom burden/health complaints (e.g., headaches, backpain). Cut-off scores identify individuals with low, medium, high, and very high somatic symptom burden. As recommended, we dichotomized the SSS-8 using 12 as the cut-off value to indicate the presence of a high or very high somatic symptom burden (<12 = low somatic symptom burden, and ≥12 = high somatic symptom burden) (Gierk et al., 2014).

Life satisfaction was assessed by the Satisfaction With Life Scale (SWLS; Diener et al., 1985). The SWLS is a 5-item scale designed to measure global cognitive judgments of one’s life satisfaction (not a measure of either positive or negative affect). Participants indicate how much they agree or disagree with each of the 5 items using a 7-point scale that ranges from 7 (strongly agree) to 1 (strongly disagree).

### Statistics

IBM SPSS version 25 (SPSS Inc., Chicago, IL, United States) for Mac was used for all analyses. Chi-square tests were used to examine possible demographical differences (sex, age, marital status, accommodation status, immigrant status, divorced parents, and financial difficulties) between students with care responsibilities and the control group (students with no care responsibilities). Number of hours spent by female and male students on care responsibilities on weekdays and weekends were also examined. Chi-square tests were used to investigate the association between hours of care responsibilities, and the prevalence of anxiety and depression, insomnia and somatic symptom burden, and life satisfaction, stratified by gender. Logistic regression analysis were conducted to provide effect-size estimates [odds-ratios (ORs)] on the same dependent variables (dichotomized), stratified by sex. The following potential confounders were included in the adjusted analyses: age, marital status, accommodation status, divorced parents, financial difficulties, and immigrant status. We also computed Estimated Marginal Means (EMM) for the three continuous outcomes measures (HSCL-25, SSS-8, and SWLS), controlling for the same confounders (not stratified by gender). Effect sizes (pooled SD) were calculated using Cohen’s d – formula. According to Cohens’ guidelines (Cohen, 1988), these effect sizes should be interpreted with ds around 0.20 representing small effect sizes, ds of about 0.50 moderate effect sizes and ds above 0.80 large effect sizes. The normality of the data was examined using skewness and kurtosis, and all continuous measures were well within the recommended ranges (±2) (George and Mallery, 2016). There was generally little missing data, and hence missing values were handled using listwise deletion. As the SHoT2018 study had several objectives and was not designed to be a study of students with care responsibilities specifically, no *a priori* power calculations were conducted to ensure that the sample size had sufficient statistical power to detect differences in outcomes.

### Ethics Statement

The SHoT2018 study was approved by the Regional Committee for Medical and Health Research Ethics in Norway (no. 2017/1176). An electronic informed consent was obtained after the participants had received a detailed introduction to the study.

## Results

### Sample Characteristics

**FIGURE 1:**
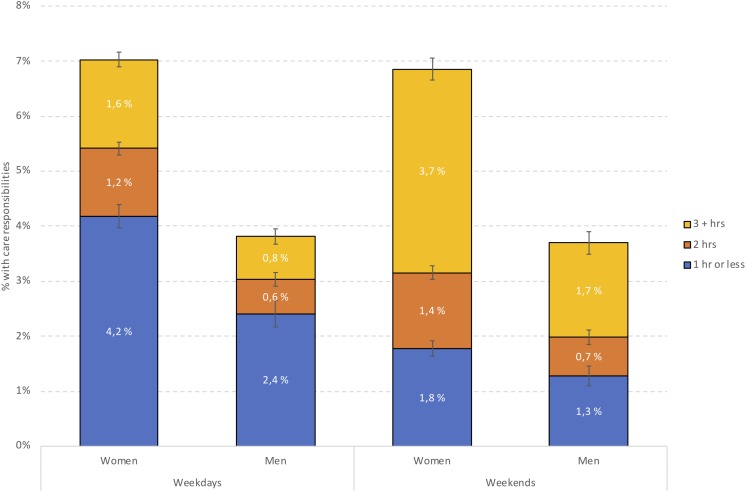
Distribution of male and female students with care responsibilities on weekdays and weekends. Note: value on left axis indicates the proportion of the whole sample, while values in bars indicate proportion among students having care responsibilities.

The sample comprised 40,205 young adults (70.2% women), with a mean age of 22 years (*SD* = 1.7). In terms of students having care responsibilities for others with physical or mental illness, disabilities, or substance misuse, 6.4% (*n* = 1804) of female and 3.4% (*n* = 416; *p* < 0.001) of male students reported this [5.5% (*n* = 2220) of the total sample]. Of these, the majority (81.3%, *n* = 1804) reported that they did not live together with the persons they had care responsibilities for. As detailed in Table 2, having care responsibilities for others was associated with being single, having divorced parents, and being of non-Norwegian ethnicity. Students with care responsibilities also reported more financial difficulties than others (see Table 2 for details). Figure 1 displays the distribution of male and female students with care responsibilities on weekdays and weekends. Both on weekdays and weekends, a significantly larger proportion of female compared to male students spent 2 h or more on care responsibilities. Correspondingly more male students spent 1 h or less on care responsibilities compared to female students (*p* < 0.001) (see Figure 1 for details). There were also significant differences between weekdays and weekends, with both female and male students spending more hours with care responsibilities on weekends than on weekdays.

**TABLE 2:** Demographical characteristics stratified by care responsibilities, with total number of young adult carers (*n* = 2220).

	**No (*n* = 37,977, 94.5%)**	**Yes, someone I live with (*n* = 536, 1.3%)**	**Yes, someone I do not live with (*n* = 1,692, 4.2%)**	***t*-value/Pearson Chi-Square (degrees of freedom)**	***p*-values**
**Hours of daily care**					
Weekdays, mean (SD)	n/a	3.34 (2.42)	2.61 (2.02)	*t* = 6.77 (2140)	<0.001
Weekend, mean (SD)	n/a	4.64 (3.35)	4.68 (3.30)	*t* = −0.22 (2083)	0.830
Sex				χ^2^ = 152.37 (2)	<0.001
Women	93,6% (*n* = 26,324)	1,4% (*n* = 399)	5,0% (*n* = 1405)		
Men	96,5% (*n* = 11,521)	1,1% (*n* = 136)	2,3% (*n* = 280)		
**Age**				χ^2^ = 20.26 (4)	<0.001
18–20 years	94,8% (*n* = 8,370)	1,4% (*n* = 125)	3,8% (*n* = 377)		
21–22 years	94,8% (*n* = 14,663)	1,4% (*n* = 209)	3,9% (*n* = 599)		
23–25 years	94,0% (*n* = 14,944)	1,3% (*n* = 202)	4,8% (*n* = 756)		
Marital status				χ^2^ = 23.01 (2)	<0.001
Single	93,9% (*n* = 17,797)	1,4% (*n* = 270)	4,7% (*n* = 887)		
Married/partner/girl- or boyfriend	95,0% (*n* = 20,154)	1,2% (*n* = 265)	3,8% (*n* = 803)		
**Accommodation status**				χ^2^ = 427.32 (6)	<0.001
Alone	94,7% (*n* = 6,472)	–	4,8% (*n* = 331)		
With partner	93,8% (*n* = 8,554)	1,5% (*n* = 133)	4,7% (*n* = 433)		
With friends/others in a collective	95,1% (*n* = 19,434)	0,9% (*n* = 191)	4,0% (*n* = 808)		
With parents	91,4% (*n* = 3,460)	5.5% (*n* = 207)	3,1% (*n* = 118)		
**Divorced parents**				χ^2^ = 191.02 (2)	<0.001
Yes	92,3% (*n* = 11,950)	1,5% (*n* = 195)	6,2% (*n* = 801)		
No	95,5% (*n* = 25,935)	1,2% (*n* = 339)	3,3% (*n* = 888)		
**Financial difficulties**				χ^2^ = 364.26 (6)	<0.001
Often	88,3% (*n* = 2,448)	2,5% (*n* = 68)	9,3% (*n* = 257)		
Sometimes	92,8% (*n* = 7,709)	1,9% (*n* = 156)	5,3% (*n* = 439)		
Seldom	94,1% (*n* = 8,661)	1,3% (*n* = 118)	4,6% (*n* = 428)		
Never	96,2% (*n* = 19,073)	1,0% (*n* = 193)	2,9% (*n* = 568)		
**Immigrants status**				χ^2^ = 38.12 (2)	<0.001
Immigrant	92,9% (*n* = 2850)	2,5% (*n* = 78)	4,5% (*n* = 139)		
Ethnic Norwegian	94,6% (*n* = 35127)	1,2% (*n* = 458)	4,2% (*n* = 1553)		

### Care Responsibilities and Mental Health Problems

**FIGURE 2:**
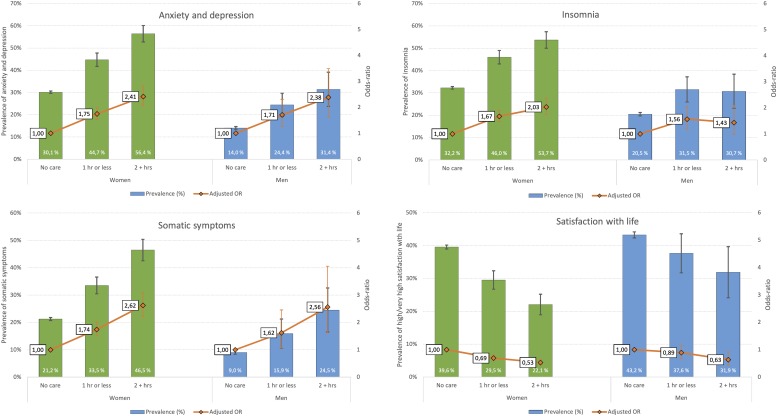
Prevalence (left axis) and odds-ratios (OR: right axis) for high level of anxiety and depression, insomnia and somatic symptoms among male and female students with care responsibilities stratified by hours of care. “Ref” indicates students with no care responsibilities (reference category). Error bars represent 95% confidence intervals of odds-ratios.

**FIGURE 3:**
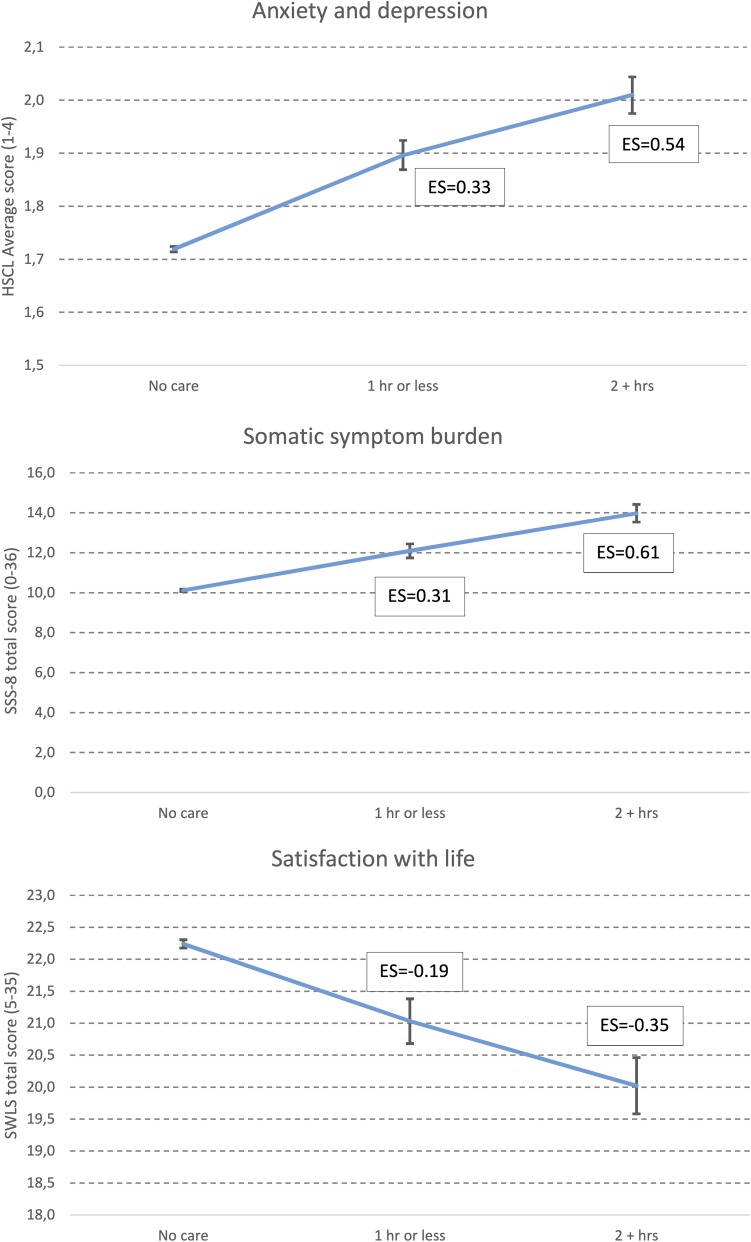
Level of anxiety and depression (HSCL-25), somatic symptom burden (SSS-8), and satisfaction with life (SWLS). Values represent Estimated Marginal Means (EMM), adjusted for age, marital status, accommodation status, divorced parents, and financial difficulties and immigrant status. Error bars represent 95% confidence intervals of odds-ratios. Text boxes represent Cohen’s d effect sizes (ES), compared with “no care”.

Mental health problems were significantly associated with amount of care responsibilities in a dose-response manner. As displayed in Figure 2, while 30.1% of female students with no care responsibilities score above the cut-off for moderate symptoms of anxiety and depression (HSCL-25 > 1.75), the corresponding proportions were 44.7% and 56.4% among women spending 1 h or less, and 2 h or more per weekday, respectively, on care responsibilities. The same pattern was observed for men, with 14% of men with no care responsibilities reporting mental health problems, compared to 24.4% and 31.4% among men spending 1 h or less, and 2 h or more per weekday, respectively, on care responsibilities. As also detailed in Figure 2 (right axis), the magnitude of associations were similar among men and women, with no significant gender differences in adjusted ORs. As displayed in Figure 3, a similar pattern was observed when analyzing the HSCL-25 as a continuous measure. Compared to individuals with no care responsibilities, the observed effect sizes were *d* = 0.33 and *d* = 0.54 for “1 h or less” and “2 or more hours” of care, respectively.

### Care Responsibilities and Insomnia

Insomnia was significantly more prevalent among students with care responsibilities. While 32.2% of female students with no care responsibilities fulfilled the DSM-V criteria for an insomnia disorder, the prevalence of insomnia was 46% and 53.7% among women spending 1 h or less, and 2 h or more per weekday, respectively, on care responsibilities. Insomnia was also more prevalent among men with care responsibilities, but the dose-response relationship observed in women was not found for men. As for mental health problems, the ORs regarding insomnia were comparable in magnitude in both genders, except for men spending 2+ h of care responsibilities (adj. OR = 1.43), which was lower than for females (adj. OR = 2.03; see Figure 2 for details).

### Care Responsibilities and Somatic Symptom Burden

A similar dose-response relationship was found between somatic symptom burden and amount of care responsibilities. While 21.1% of female students with no care responsibilities were classified as having a high or very high somatic symptom burden, the corresponding proportions were 33.5% and 46.5% among women spending 1 h or less, and 2 h or more per weekday, respectively, on care responsibilities. The same pattern was observed for men, with 9% of men with no care responsibilities reporting a high or very high somatic symptom burden, compared to 15.9% and 24.5% among men spending 1 h or less, and 2 h or more per weekday, respectively, on care responsibilities. No significant differences were observed between men and women regarding the strengths of associations (adj. ORs). A similar pattern was observed when analyzing the total score of the SSS-8. Compared to individuals with no care responsibilities, the observed effect sizes were *d* = 0.31 and *d* = 0.61 for “1 h or less” and “2 or more hours” of care, respectively.

### Care Responsibilities and Satisfaction With Life

An inverse dose-response relationship was observed between life satisfaction and amount of care responsibilities. While 39.6% of female students with no care responsibilities reported high or very high life satisfaction, the corresponding proportions were 29.5% and 22.1% among women spending 1 h or less, and 2 h or more per weekday, respectively, on care responsibilities. The same pattern was observed for men, with 43.2% of men with no care responsibilities reporting a high or very high life satisfaction, compared to 37.6% and 31.9% among men spending 1 h or less, and 2 h or more per weekday, respectively, on care responsibilities. The adjusted ORs were somewhat weaker for men compared to women (see Figure 2 for details). A similar pattern was observed when analyzing the total score of the SWLS. Compared to individuals with no care responsibilities, the observed effect sizes were *d* = −0.19 and *d* = −0.35 for “1 h or less” and “2 or more hours” of care, respectively.

## Discussion

In the national student survey (*N* = 40,203, 18–25 years) 5.5% of the respondents reported that they had care responsibilities for family members or others (not including own children) with physical or mental illness, disabilities, or substance misuse. Caring was associated with being female, single, having divorced parents, being immigrant, and having financial difficulties. The majority of students with care responsibilities did not live with the person they cared for. They spent more time during weekends compared to weekdays on care responsibilities, with around 50%, both men and women, spending 3 h or more on caring responsibilities per day on weekends. For both men and women, more mental health problems, insomnia, and somatic symptoms, as well as lower satisfaction with life, were found among students with care responsibilities, compared to students without care responsibilities. The number of hours spent on caring was associated with the magnitude of mental health problems, insomnia, somatic symptoms and satisfaction with life in a dose-response pattern.

### Prevalence and Gender Differences

Few large-scale surveys provide estimates on prevalence of caregiving among young adults. However, a study in United Kingdom identified 5.3% of young adults aged 18–24 as carers (Becker and Becker, 2008), almost identical to the prevalence in the present study. Furthermore, by reviewing estimates of young carers (between 10 and 24 years) in 7 studies from different European countries, Leu et al. (2019) found prevalence rates ranging from 4.5% to 8%. According to the national statistical institute there are about 545 000 young adults between 18 and 25 years in Norway (StatisticsNorway, 2019). With an estimate of 5.5% YAC, just below 30 000 young adults, on a national level, are assumed to care for chronically ill, substance misusing or disabled family members or others.

The prevalence of caregiving in the current study was significantly higher in women (6.4%) compared to men (3.4%). Furthermore, on average, the female students spent more hours on caregiving than male students did. Similar gender difference has been reported in other age groups of caregivers, i.e., older adults, children, and adolescents (Nagl-Cupal et al., 2014; Berglund et al., 2015; Chikhradze et al., 2017; Leu et al., 2019), whereas other studies report higher prevalence of male caregivers (Levine et al., 2005; Pakenham and Cox, 2015). The mixed findings between studies may be a result of different recruitment strategies, samples and measurements. It has for example been found that females are more involved in some type of caring activities, such as domestic work, than others (Joseph et al., 2019). If the findings in the current study are replicated, we need to examine why the responsibility for care lay more heavily on young adult women. Exploring differences in gender roles, cultural expectations, values and priorities among men and women could contribute to a better understanding of the gender differences in informal caregiving.

### Financial Stress, Family Structure, and Migrant Families

Young adult carers reported more financial difficulties compared to other students. This is in line with previous findings (Becker and Becker, 2008). Financial hardship among YACs may be a consequence of low income in families with one or more family member with chronically illness, substance misuse or disability. However, the financial difficulties reported by YACs may also be a result of conflicting demands of combining care responsibilities, education and part-time employment (Becker and Becker, 2008). For some of YACs there might just not be enough time to take on a part-time job besides studies and care responsibilities.

Higher prevalence of caregiving was found among students from divorced families. This finding is in line with previous studies (Ireland and Pakenham, 2010; Pakenham and Cox, 2015; Chikhradze et al., 2017). Associations between having divorced parents and care responsibility may be understood as a vulnerability in single-parent families. In divorced families, the young adult may have less choice about becoming a caregiver, especially if the single parent is the one who is the care-recipient, and if no other healthy parent is present in the family to share the care responsibility.

Our results indicated that more students from immigrant families provide informal care. In a report from four European countries on the situation for young ethnic minority carers below 30 years, higher prevalence of carers in migrant families is assumed to be the result of several mechanisms (Care2Work, 2017). Migrant families may have lower awareness of how the welfare state works and where to turn for help and may therefore be less likely to access services that support people with a disability or chronic illness. They may also have stronger culturally attitudes of shame or stigma associated with disability or mental illness. Additionally, some may have language barriers, making it difficult to access the help they need. Finally, there may be higher cultural expectations that care should be provided by family members, and the young adults may themselves experience a strong moral obligation to take care of family members in need (Care2Work, 2017).

We have presented demographic characteristics of YACs. These suggest that some young adults (e.g., immigrants and young adults from divorced families) may be more willing to or perhaps have less choice about taking on care tasks. In immigrant families, as well as single parent families there may not be sufficient income to pay for external help. There may not be others present to provide the care needed, there may be no community care or home-based services available, or if available, it may not be acceptable for family members to receive these services (Day, 2015; Chikhradze et al., 2017).

### Negative Health Outcomes and Amount of Care Responsibilities

In the present study, students who confirmed caregiving responsibility had more negative health outcomes compared to non-caregiving students. Whereas previous studies have found that young carers experience the caring responsibility as rewarding (Chikhradze et al., 2017), carers in different age groups also report adverse effects (Pakenham et al., 2006; Berglund et al., 2015; Pakenham and Cox, 2015; Greene et al., 2017; Kallander et al., 2018). In line with increased rate of health problems found among carers in general (Koyanagi et al., 2018), the negative outcomes among YACs in the present study were evident on several health markers, i.e., anxiety and depressive symptoms, sleep problems, and somatic symptoms. These results should, however, be understood in the context of the transition period of emerging adulthood (e.g., leaving home, starting higher education) (Arnett, 2007). It has been suggest that while the lives of non-caregiving emerging adults reach a peak of new-found autonomy and possibility, the lives of YACs reach a peak of dismay and isolation (Becker and Becker, 2008). Increased rates of anxiety and depressive symptoms, insomnia and somatic complaints reported by YACs may reflect emotional stress responses of worrying, loneliness and guilt related to the role as carer, as well as possible shame, anxiety and worry associated with the illness, substance misuse or disability of the person they are caring for Chikhradze et al. (2017). Furthermore, YACs may also have emotional reactions due to the caring responsibilities leaving them with limited time for relaxation, social life and leisure activities.

The dose-response association found between extent of caring and negative health outcomes, suggests that the adverse effects of caring increase parallel to the hours invested in looking after the care-recipient. According to a previous study, YACs become vulnerable when the level of care-giving becomes excessive (Becker and Becker, 2008). The negative outcomes observed among YACs may be a result of the pressure of managing education, personal relationships and the hours needed to care for the ill or disabled relative. However, more time-consuming caring might also be an indicator of how ill or disabled the care-recipient is, the amount of social resources available in the family, and/or the lack of help and support received from health and/or social services. According to the current study, negative health outcomes occur also after the young person has left the family of origin to live elsewhere. It is possible that this may be a result of continuing anxiety, stress, tiredness and physical and emotional strain associated with the caring-roles of YACs.

### Satisfaction With Life and Amount of Care Responsibilities

Positive outcomes of caring is emphasized in the literature (e.g., Winton, 2003; Haugland, 2006; Becker and Becker, 2008). We included satisfaction with life as a positive outcome measure to assess beneficial effects of care responsibility. However, the results indicate lower life satisfaction in YACs compared to other students. Furthermore, lower life satisfaction was associated with higher number of hours spent on care responsibilities. This suggests that life satisfaction may not capture the positive consequences of providing care for someone close. Probably other measures are needed to capture the characteristics that have been suggested as positive outcomes of caregiving, e.g., increased maturity, autonomy, sensitivity, empathy, and life skills (Winton, 2003; Becker and Becker, 2008).

### Strengths and Limitations

The strengths of the present study include a large study population and psychometrically sound measures. The survey questions applied to identify YACs have been thoroughly examined for clarity (Cheesbrough et al., 2017b), and assumed to be suitable for identifying young carers.

The results should be interpreted in accordance to the relatively modest response rate for the survey (31%), with little information about the characteristics of non-participants beyond age and gender distribution. The prevalence estimate of YACs from this study is based on self-report. Because young carers are often not identified by professionals in health care, education and social services (Leu et al., 2018), self-report measures are commonly used and considered the best available strategy to identify this group of cares. However, no information about or definition of YACs was provided to the responders in the survey. We assume that the awareness of the role of young carer is limited among Norwegian students. This may have made it more difficult for the students to recognize the care responsibility they are providing. This would represent a bias toward an underestimation of the prevalence of YACs in the present study.

Young carers may experience barrier against entering higher education, e.g., due to inability to leave the family or the person they care for and insufficient support and guidance at school (Becker and Becker, 2008). This may be especially true when it comes to young carers from ethnic minorities (Care2Work, 2017). Thus, a selection bias might be present in our sample, probably resulting in a lower estimated prevalence of YACs. Due to this potential selection bias, the present results should not be generalized to the whole population of YACs.

As females constitute about 70% of the student population in Norwegian colleges/universities, the gender difference in the sample should not represent a substantial bias in the current study. Other limitations include the lack of information about the type of care tasks performed, whether the young person cares for someone with physical or mental health problems, disability, or substance misuse, and whether the care recipient is a parent, a sibling, a friend or a partner. As the focus of the present study was mainly on negative health consequences, possible beneficial effects of the caregiving role beyond life satisfaction were not included. This is an important limitation as caregiving has been found to also have positive emotional and psychological benefits for the carer (Cheesbrough et al., 2017a). Being a cross-sectional study we cannot determine the temporal order and causality between caring responsibility and health outcomes. However, caregiving most likely affects health outcomes, rather than the other way around.

### Implications

Young adults who care for ill, substance misusing or disabled relatives or others need to be acknowledged and to receive targeted support. These young adults are a great resource for family members, for the health care system and for society, and their willingness to provide care should be recognized and valued. Lack of practical, emotional and financial support may be related to health problems and reduced life opportunities for YACs. When family members are chronically ill, disabled or substance misusers, it is essential that the health consequences of *all* family members is considered, including the situation for the young adults who no longer live in the family household. This is increasingly important, as the need for informal care is expected to rise in the future, due to more outpatient care for patients with chronic illnesses, increasing number of single parent households, and a growing population of older persons. To develop interventions to support YACs and prevent negative health consequences, greater awareness among politicians and decision makers in social services, community planning, and education is warranted.

Our results show that interventions should address ways to reduce the hours needed for YACs to provide care, preferably by providing flexible help for families from home-based services. As the negative health outcomes are related to hours of caring, support that reduce the care responsibility seems to be particularly important. This may also make it easier for YACs to achieve their educational goals.

Young adult carers report that they need someone to talk to, someone who may offer hope, give advice and with whom they can share experiences and coping strategies (Ali et al., 2013). Developing adequate interventions (e.g., support groups, networks, and web-support) may prevent or moderate negative health consequences among YACs. However, these need to be delivered with respect and sensitivity, to overcome potential barriers of fear, shame and loyalty that may make it difficult for many YACs to seek external support (Ali et al., 2013).

### Conclusion

Young adult carers in higher education in Norway have more negative health problems (i.e., symptoms of anxiety and depression, sleep problems, and somatic symptoms), compared to other students. This vulnerability needs to be acknowledged by Norwegian authorities and professionals within health care, social services, and the educational system. We need to develop interventions that support YACs who struggle to balance life between caring, completing education, and fulfilling personal and social aims. In addition, home-based services should be available for families with chronically ill, substance misusing and disabled person. The small number of research studies on YACs internationally indicate that there may be limited awareness about the health and educational consequences of caring in many countries. The burden of caregiving needs to be considered when investigating health problems among young adult students across countries.

## Data Availability Statement

The datasets for this article are not publicly available because of privacy regulations from the Norwegian Regional Committees for Medical and Health Research Ethics (REC). Requests to access the datasets should be directed to BS (borge.sivertsen@fhi.no). Guidelines for access to SHoT data are found at https://www.fhi.no/en/more/access-to-data. Approval from REC (https://helseforskning.etikkom.no) is a pre-requirement.

## Ethics Statement

The studies involving human participants were reviewed and approved by the Regional Committee for Medical and Health Research Ethics in Western Norway (no. 2017/1176). The patients/participants provided their written informed consent to participate in this study.

## Author Contributions

BH, MH, and BS contributed to the conception of the present study. BS performed the statistical analyses. BH and MH participated in the interpretation of the data. BH and BS wrote the first draft of the manuscript. All authors contributed to the manuscript revision, and read and approved the submitted version.

## Conflict of Interest

The authors declare that the research was conducted in the absence of any commercial or financial relationships that could be construed as a potential conflict of interest.
